# Draft Genome Sequence of *Salmacisia buchloëana* (Basidiomycota), Which Induces Hermaphroditism in Dioecious Buffalograss

**DOI:** 10.1128/genomeA.00142-17

**Published:** 2017-04-06

**Authors:** David R. Huff, Tom Hsiang, Ambika Chandra, Yu Zhang

**Affiliations:** a Department of Plant Science, Pennsylvania State University, University Park, Pennsylvania, USA; b School of Environmental Sciences, University of Guelph, Guelph, Ontario, Canada; c Texas A&M AgriLife Research and Extension Center, Dallas, Texas, USA; d Intercollege Graduate Degree Program in Plant Biology, Huck Institute of Life Sciences, Pennsylvania State University, University Park, Pennsylvania, USA

## Abstract

Here, we report the draft genome of *Salmacisia buchloëana* type strain OK1, a monotypic species of fungi that induces ovary development in genetic male plants and increases reproductive biomass allocation in its host buffalograss. This research will enhance our understanding of fungal manipulation of host development at the genomic level.

## GENOME ANNOUNCEMENT

*Salmacisia buchloëana* (Kellerman and Swingle 1889) Huff and Chandra 2008 is the only species within the genus *Salmacisia* and represents a sister lineage to grass-infecting bunt fungi within the genus *Tilletia* (1). *S. buchloëana* is an endoparasitic fungus that induces hermaphroditism in its dioecious host buffalograss (*Bouteloua dactyloides* [Nutt.] Columbus) (1). The most striking symptom of *S. buchloëana* infection is the induction of female reproductive organs (stigma, style, and ovary) in flowers of unisexual male plants, which are genetically programmed to inhibit the development of these structures (1, 2). Infection by *S. buchloëana* also alters the flowering process of its host by significantly increasing reproductive biomass allocation and seed yield components, for example, inflorescences per plant and florets per spikelet (3). *S. buchloëana* likely gains an evolutionary advantage for increasing its host’s total ovary production because it parasitizes host ovaries in order to complete its life cycle. We speculate that a potential coevolutionary process between *S. buchloëana* and its host may have resulted in numerous fungal effector genes that regulate host meristem determinacy and floral transcription factor pathways (4). Discovering these underlying *S. buchloëana* regulatory mechanisms would provide insight into the evolution of host-manipulating parasites and potentially provide new strategies for increasing seed yield in perennial grasses.

Strain OK1 (WSP_71313 holotype; PAC_106969 isotype), the type strain of *S. buchloëana*, was originally isolated in 1986 (5) and subsequently found to be culturable on potato dextrose agar. Genomic DNA was extracted from OK1 using the Qiagen DNeasy plant minikit, and submitted to the Huck Institute’s Penn State Genomic Core Facility for fragmentation, library preparation, and sequencing on the Illumina MiSeq platform. Library inserts were 550 bp and sequencing was paired-end 250 bp. The resulting 15 Gb of raw sequences were processed and assembled using different *k*-mers to generate over 20 assemblies from each of three different assembly programs—SOAPdenovo version. 1.1.2, ABySS version 1.2.3, and Velvet version 1.0.12—following Haridas et al. (6) and Li et al. (7). The highest *N*_50_ assembly (*N*_50_ = 363,681 bp, at *k*-mer = 55) was assembled with SOAPdenovo and finished with GapCloser version 1.12, resulting in a draft genome of 18.42 Mb contained in 331 contigs with a G+C content of 59.3%. AUGUSTUS version 3.2.2 *ab initio* gene predictions, based on the *Ustilago maydis* gene model for training, yielded 6,262 predicted genes with a median gene size of 1,975 bp and an average G+C content of 63.5%. This draft genome sequence of *S. buchloëana* strain OK1 contains 96.37% (235 complete single-copy and four fragmented) of the 248 CEGMAs (8) and 96.17% (1,277 complete single-copy and 106 fragmented) of the 1,438 fungal BUSCOs (9).

Understanding fungal manipulation of host development is a major goal of biotrophic interaction research (10). The purpose of sequencing the *S. buchloëana* genome was to gain insight into its gene content and organization in order to enhance our understanding of *S. buchloëana*’s remarkable ability to regulate the growth, development, and sexual expression of its perennial grass host.

### Accession number(s).

This whole-genome shotgun project has been deposited at DDBJ/ENA/GenBank under the accession number MOEQ00000000. The version described in this paper is version MOEQ01000000.
